# Annotations

**Published:** 1903-05-16

**Authors:** 


### Annotations.

### The Physiology of Shakespeare.

In the play of "Coriolanus" and in the passage where the stomach is described as being put upon its defence, the following passage occurs :

True is it, my incorporate friends, quoth he,
That I receive the general food at first,
Which you do live upon ; and fit it is,
Because I am the store-house and the shop
Of the whole body ; but if you do remember,
I send it through the rivers of your blood,
Even to the court, the heart, to the seat o' the brain,
And through the cranks and offices of man ;
The strongest nerves and small inferior veins
From me receive that natural competency
Whereby they live.

Considering the date at which it was written and the curious conceptions in vogue at the time of most life processes, this passage has always seemed to us noteworthy. But for the ascription to the stomach of a virtue which might more properly be claimed by the glycogen-forming liver, it might almost have been written yesterday by a lecturer on physiology with a turn for metrical expression. In quoting it in a discussion on the "Medical Knowledge of Shakespeare" in the *Westminster Review*, Dr. John Knott reads "veins of blood," but "rivers of your blood" is the reading in all the texts with which we are acquainted, and also preferable, because no confusion between the functions of veins and arteries is thereby introduced. Another passage quoted by Dr. Knott is a reference to the callus thrown out by a broken bone, and the thickening and apparent strengthening of the latter after fracture.

Our peace will, like a broken limb united,
Grow stronger for the breaking.

There are also a good many expressions and sentences to be discovered by the medical Shakesperean student which seem to indicate that Shakespeare had at least some premonition of Harvey's discovery of the circulation of the blood. Instances of them, quoted by Dr. Knott, occur in "Julius Cæsar," Act 2, Scene 1; "Hamlet," Act 1, Scene 5; "Measure for Measure," Act 2, Scene 4; and "Henry IV.," Part 2, Act 4, Scene 3. In this latter play, it is to be noted, there is also a very accurate description of the typical appearances of age. The "dry hand," the "yellow cheek," "decreasing leg, increasing belly," "broken voice and short wind." In the early part of "Henry V." there is also a good clinical picture of what commonly occurs in the last hours of a fatal fever, the pinched features, circulation gradually failing at the extremities, plucking at the bed clothes and visionary smile.

### The Victorian Strike.

The worst enemy of State control of industry could hardly have invented anything so well calculated to damage its popularity as the strike of the Victorian railway men. These men are civil servants, possessing all the advantages of security of tenure and guarantee of payment which that title implies, and being correspondingly bound to honest and faithful service. Yet they want to be free to join a political and Trade-union association which may at any moment call them out, in sympathy with other workers, although they have no grievance of their own. In short, they want to serve two masters. That the Australian Government should refuse their servants permission to do this is only reasonable, although, when one recalls how the Government has so long been carried on for the benefit of the so-called working classes, to the detriment of the colony as a whole, it is possible that the firm attitude now shown may surprise the strikers. But the situation proves clearly that State control does not work for efficiency and economy to the extent claimed for it by its advocates. Indeed, this strike may bring before people's minds the possible dangers to the welfare of the whole community of a state of affairs in which there would be only one employer, even though that employer were the State, since it is evident that the State cannot, any more than a private master, completely control its employés. At present the competition between rival masters and men keeps strikes from being more than partial. When one set refuse to work, the other men in the same trade are the more sure of steady work and good wages. And between the rival forces the community at large manages to get a sufficiency of service. But if everything were centralised this advantage would disappear, and we might at any moment be deprived of every necessary of life, every convenience that was under the control of the State, because one set of the State workers had a real or fancied grievance. Individualism is not popular in these days, but at least individualism saves the community from being at the mercy of its own servants.

### Paper versus Linen.

In spite of prejudice, the Japanese paper serviette is making headway, and perhaps it would become even more popular if it were not for its brilliant decoration, which rather jars upon the taste of people who associate the refinements of the table with spotless white linen. But the paper handkerchief has even greater claims upon our approbation. A handkerchief which is merely an ornamental adjunct to a lady's toilette is all very well, but there is no innate daintiness about the *mouchoir* and its uses. The washing of one which has seen the service for which it was intended is by no means a task for the fastidious, and with our increasing knowledge of the spread of disease, it is a question if it is not a reasonable sanitary precaution that all such should be destroyed. If that idea once got into the public mind, the future of the paper handkerchief should be assured. It is portable, it is not unpleasant to use, it is sanitary, and it is cheap. At the price of a farthing apiece, four of them do not cost more than the price of laundrying a cambric handkerchief, and there is the original cost of the linen in addition. Besides, as everyone knows, our handkerchiefs have a mysterious way of vanishing at the wash. Therefore the paper *mouchoir*, which can be burned when done with, appeals to us on the score of economy as well as that of convenience. The convenience of them for travellers is obvious, and perhaps through them they will work their way into general use, as other handy articles have done. But a little recommendation from sanitarians, and a little advertisement from leaders of fashion, would be a great help towards what our grandchildren will doubtless think a most obvious step in the direction of sanitary living.

